# Retraction Note: LncRNA LINC00261 overexpression suppresses the growth and metastasis of lung cancer via regulating miR-1269a/FOXO1 axis

**DOI:** 10.1186/s12935-024-03565-1

**Published:** 2024-11-09

**Authors:** Caixia Guo, Hongmei Shi, Yuli Shang, Yafei Zhang, Jiajia Cui, Hongtao Yu

**Affiliations:** Department of Respiratory Medicine, Henan Provincial Chest Hospital, No.1, Weiwu Road, Zhengzhou, 450000 Henan Province China

**Cancer Cell International (2020) 20:275**

10.1186/s12935-020-01332-6

The Editors-in-Chief have retracted this article at the corresponding author’s request. After publication, concerns were raised regarding highly similar colony formation assay images between Fig. 8e in this article and a number of other articles from other groups [1–4]. The authors have been unable to provide satisfactory raw data to address these concerns.

The Editors-in-Chief therefore no longer have confidence in the presented data.

Hongtao Yu agrees with this retraction. None of the other authors have responded to correspondence from the Publisher about this retraction.
